# Physical Activity Participation Decreases the Risk of Depression in Older Adults: The ATHLOS Population-Based Cohort Study

**DOI:** 10.1186/s40798-023-00664-7

**Published:** 2024-01-03

**Authors:** Rodrigo A. Lima, Elena Condominas, Albert Sanchez-Niubo, Beatriz Olaya, Ai Koyanagi, Carlota de Miquel, Josep Maria Haro

**Affiliations:** 1 Research, Innovation and Teaching Unit, Parc Sanitari Sant Joan de Déu, Dr Antoni Pujadas 42, 08830 Sant Boi de Llobregat, Spain; 2 Centro de Investigación Biomédica en Red de Salud Mental (CIBERSAM), Instituto de Salud Carlos III, Madrid, Spain; 3 Department of Social Psychology and Quantitative Psychology, University of Barcelona, Barcelona, Spain

**Keywords:** Depression, Longitudinal analysis, Physical activity, Data harmonisation, Health metric, Lifestyle behaviours

## Abstract

**Background:**

To which extent physical activity is associated with depression independent of older adults’ physical and cognitive functioning is largely unknown. This cohort study using harmonised data by the EU Ageing Trajectories of Health: Longitudinal Opportunities and Synergies consortium, including over 20 countries, to evaluate the longitudinal association of physical activity (light-to-moderate or vigorous intensity) with depression in older adults (aged ≥ 50 years).

**Results:**

We evaluated 56,818 participants (light-to-moderate models; 52.7% females, age 50–102 years) and 62,656 participants (vigorous models; 52.7% females, age 50–105 years). Compared to never, light-to-moderate or vigorous physical activity was associated with a lower incidence rate ratio (IRR) of depression (light-to-moderate model: once/week: 0.632, 95% CI 0.602–0.663; twice or more/week: 0.488, 95% CI 0.468–0.510; vigorous model: once/week: 0.652, 95% CI 0.623–0.683; twice or more/week: 0.591, 95% CI 0.566–0.616). Physical activity remained associated with depression after adjustment for the healthy ageing scale, which is a scale that incorporated 41 items of physical and cognitive functioning (light-to-moderate model: once/week: 0.787, 95% CI 0.752–0.824; twice or more/week: 0.711, 95% CI 0.682–0.742; vigorous model: once/week: 0.828, 95% CI 0.792–0.866; twice or more/week: 0.820, 95% CI 0.786–0.856).

**Conclusions:**

Physical activity, of any intensity and weekly frequency, was a strong protective factor against depression, independent of physical and mental functioning. Health policies could stimulate the incorporation of lower physical activity intensity to protect against depression, which might be more feasible at the population level.

**Supplementary Information:**

The online version contains supplementary material available at 10.1186/s40798-023-00664-7.

## Key Points

- Independent of older adult’s physical and mental functioning, physical activity of any intensity (light-to-moderate or vigorous) and weekly frequency (once/week or higher frequency) conferred strong protection against the risk of depression in older adults
- Public health policies should stimulate the incorporation of lower physical activity intensities to prevent depression in older adults, which might be more feasible at the population level.

**Supplementary Information:**

The online version contains supplementary material available at 10.1186/s40798-023-00664-7.

## Background

Depression is the leading cause of mental health-related disease burden globally and affects approximately 300 million people worldwide [1, 2], explaining 5.6% of all years lived with disability globally—being the second highest in the rank [3]. Because of this societal burden, the scientific community has sought to identify relevant factors that decrease the risk of depression in the adult population.

Systematic reviews and meta-analyses reported a lower risk of incident depression or odds of presenting depression for adults and older adults who perform physical activities. The risk of incident depression has been estimated to be 16–31% lower for adults [4–6] and 21% lower for older adults [4, 6] who participate in physical activities, especially in moderate-to-vigorous intensities. Furthermore, higher time in moderate-to-vigorous physical activity was associated with a 12–32% lower chance of presenting depressive symptoms during the COVID-19 pandemic [7].

Pearce et al. [8] evaluated the dose–response relationship of physical activity with risk of depression by assessing 15 studies comprising 191,130 individuals. Relative to adults not reporting any activity, adults who accumulated half the recommended volume of physical activity per week exhibited lower risk of depression by 18%, whereas the risk was 25% lower for adults accumulating the recommended 8.8 marginal metabolic hours per week of physical activity. Furthermore, 11.5% of depression cases could have been prevented if less active adults met physical activity recommendations [8].

Despite the abovementioned wealth of evidence, there are a number of limitations in the state-of-the-art, diminishing the applicability and extension of the findings. To date, studies have considered several factors that might confound the relationship between physical activity and depression, such as sex, age, alcohol use, smoking or physical health status [4–6, 8]. However, population-level factors are difficult to measure and it is thus possible that previous studies suffered from residual confounding.

Particularly in older adults, it is pivotal to consider their health status while evaluating the association of physical activity with depression, hence estimating whether physical activity protects against depression independent of health status. Nevertheless, often, studies only include older adults’ health perception or physical or cognitive functioning [4–6, 8], which is relevant, but likely not reflecting their health status holistically.

We intended to overcome these limitations by using data from the EU Ageing Trajectories of Health: Longitudinal Opportunities and Synergies consortium—the ATHLOS project cohort. First, ATHLOS contains harmonised data from different international cohort studies covering different countries and continents [9]. In particular, results derived from the integration of harmonised individual data, such as in the ATHLOS cohort, may lead to greater validity compared to ‘standard’ systematic reviews and meta-analyses [9–12]. The ATHLOS cohort allows for more direct comparison between subjects from different studies, increasing the applicability of the findings to a wider audience [9].

Second, the ATHLOS project constructed and validated the ATHLOS healthy ageing scale [13], which is based on 41 parameters covering domains of vitality, sensory functions, locomotion, cognition and activities of daily living from seven domains [13] (see Additional file 1: Table S1). This scale was intended to capture the concepts of intrinsic capacity and functional ability as defined by the WHO [14]. Considering the ATHLOS healthy ageing scale in the evaluation of the physical activity–depression relationship is a unique step forward in the field.

A previous study from the ATHLOS consortium showed that engagement in vigorous or lower physical activity intensity was associated with lower odds of presenting a fast decline in the healthy ageing scale or being in the group of adults with a continuous low level of healthy ageing [15]. Nevertheless, it is not clear whether or to which extent physical activity would be associated with depression after considering a comprehensive healthy ageing scale, such as ATHLOS.

### Objective

Therefore, we evaluated the longitudinal association of physical activity (light-to-moderate and vigorous intensities) with depression, and whether the ATHLOS healthy ageing score influenced the relationship between physical activity and depression.

## Methods

### Study Design and Population

This is a longitudinal study using data from the ATHLOS cohort [9, 13], which consists of 17 ageing studies across the world and harmonised a wide range of lifestyle, social, environmental, physical and psychological health factors. Documentation of the harmonisation process is available online [9, 13]. All cohort studies obtained approval from the respective local research ethics communities, and informed consent from the participants.

For the present study, we used three studies (HRS: Health and Retirement Study; SHARE: Survey of Health Ageing and Retirement in Europe; and, KLOSA: Korean Longitudinal Study) that contained data on physical activity and depression in at least two waves. Additional file 1: Fig. S1 presents additional information on the studies included besides the timeline of the cohort studies and respective waves that contributed with data to the current investigation.

**Fig. 1:**
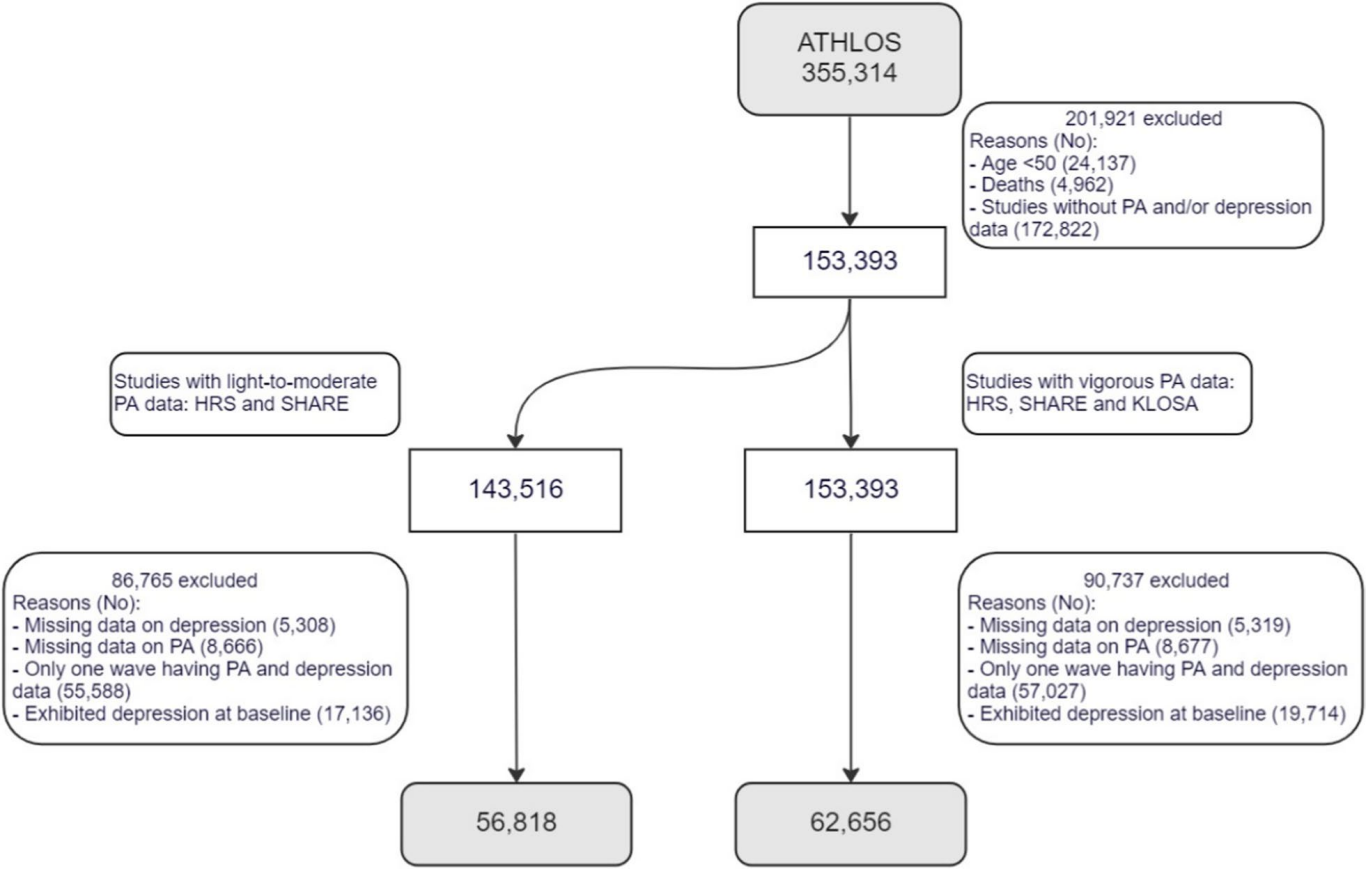
Flow diagram of the data selection process

Figure 1 shows the flowchart of participants. From the 355,314 participants in ATHLOS, 201,921 were not eligible: 24,137 < 50 years, 4962 deaths and 172,822 were in studies without physical activity and depression data. From the 153,393 eligible participants, 62,656 (vigorous physical activity) and 56,818 (light-to-moderate physical activity) were included in the current study.

### Measures

#### Depression

Before harmonisation, current depression status was assessed by the Depression Scale of the Center for Epidemiological Studies (CES-D) with 8 items in the HRS study and with 10 items in the KLOSA study, whereas the SHARE study used the EURO-D (European Union initiative to compare symptoms of depression) scale. Individuals who presented four or more clinical symptoms (HRS and SHARE studies) or 10 or more symptoms (KLOSA study) were classified with depression, following standard protocol for each of the questionnaires [16].

#### Physical Activity

We used two harmonised variables for physical activity: frequency of light-to-moderate physical activity and frequency of vigorous physical activity during the last week.

The weekly frequency of light-to-moderate physical activity was computed by the following questions; SHARE study: “How often do you engage in activities that require a low or moderate level of energy such as gardening, cleaning the car, or doing a walk?”; HRS study: “How often do you participate in light physical activity—such as walking, dancing, gardening, golfing, bowling, etc.?”.

The weekly frequency of vigorous physical activity was computed by the following questions; SHARE study: “How often do you engage in vigorous physical activity, such as sports, heavy housework, or a job that involves physical labour?”; and HRS and KLOSA studies "How often do you participate in vigorous physical exercise or sports—such as aerobics, running, swimming, or bicycling?”.

Because HRS, KLOSA and SHARE recorded physical activity participation differently (number of times/week), we classified the participation into light-to-moderate and vigorous physical activity in two levels of detail: (1) never, once a week and twice or more a week—HRS, KLOSA and SHARE studies; and (2) never, once a week, 2–3 days a week and more than three days a week—HRS and KLOSA studies.

#### Healthy Ageing Scale

The ATHLOS healthy ageing scale was constructed by using a two-parameter logistic item response theory (IRT) model with 41 items related to intrinsic capacity and functional ability. A detailed description of the creation of the ATHLOS scale is available elsewhere[13]—see also Additional file 1: Table S1. The scale scores are normally distributed with a mean of 50 and a standard deviation of 10, with higher values indicating more favourable levels of healthy ageing. The healthy ageing score was divided into quartiles (25th Percentile: 45.5; 50th Percentile: 52.7; 75th Percentile: 59.4) using the three studies included.

#### Confounders

Sex and age were inquired in each of the original cohort studies. Total household wealth in quintiles was calculated within cohorts, with the highest quintile being the most prosperous. Education was divided and harmonized into primary, secondary or tertiary based on the ISCED-2011 scale. Harmonised current smoking status (yes and no) and current alcohol drinking status (yes and no) were also included.

### Statistical Analyses

All statistical analyses were conducted with STATA 16 for windows (StataCorp LP, College Station, TX, USA). We accepted 5% type I error in all analyses. Descriptive statistics are presented in means and standard deviations or in absolute and relative frequencies.

We applied Poisson generalised estimating equation (GEE) mixed model with exchangeable correlation structure and a robust error variance correction to evaluate the longitudinal association of light-to-moderate and vigorous physical activity participation (never, once a week and twice or more a week; exposure) with incident depression (yes or no; outcome), presented as incidence rate ratio (IRR). Of note, different models were constructed for each physical activity intensity, and all participants included in the current study did not have depression at baseline.

In total, four models were built for the main analyses. Initially, a Poisson GEE mixed model included physical activity (light-to-moderate or vigorous intensity) as the exposure and depression as the outcome adjusted by the confounders (sex, age, education level, wealth, current smoking, current alcohol drinking and cohort study). Then, the same models described above also adjusted by the participants’ healthy ageing status. We used the QIC (Quasi-Information Criterion) for assessing the adequacy of the GEE models, particularly the correlation information criterion parameter (CIC), in which the model with the lowest CIC value should be prioritised compared to models with higher CIC [17].

Additional information on the GEE mixed models applied can be found in Additional file 1.

To complement the main analysis, we reran the same four models with only one difference: physical activity (light-to-moderate and vigorous intensities) participation contained four possible frequencies of physical activity participation (never, once a week, 2–3 days a week and more than three days a week) instead of three (never, once a week and twice or more a week). These complementary analyses were conducted with participants from HRS and KLOSA cohorts because SHARE did not classify weekly physical activity participation in four categories.

#### Sub-group Analysis

We conducted the Poisson GEE mixed models described previously for the main analysis stratified by sex (males and females), age group (50–59, 60–69, 70 + years), study (HRS, KLOSA and SHARE) and the ATHLOS healthy ageing status at baseline (1st–4th quartile) to investigate whether these variables moderated the association between physical activity and depression.

## Results

We evaluated 56,818 participants (light-to-moderate models; 52.7% females, age 50–102 years), and 62,656 participants (vigorous models; 52.7% females, age 50–105 years). Table 1 presents the demographic characteristics of the participants at baseline. Additional file 1: Table S2 presents the demographic characteristics of participants included in the current study at baseline compared to the total in the ATHLOS cohort.

**Table 1:** Baseline characteristics of the study population with information on light-to-moderate and vigorous physical activity

Characteristics	Light-to-moderate PA models, No (%)			Vigorous PA models, No (%)			
	HRS^a^	SHARE^b^	Total included	HRS	KLOSA^c^	SHARE	Total included
	No = 19,187	No = 37,631	No = 56,818	No = 19,170	No = 5857	No = 37,629	No = 62,656
	USA	Europe		USA	South Korea	Europe	
*Age, means (SD), y*	63.7 (10.3)	63.2 (9.3)	63.4 (9.6)	63.7 (10.3)	61.0 (9.6)	63.2 (9.3)	63.2 (9.6)
*Sex*							
Female	10,829 (56.4)	19,135 (50.8)	29,964 (52.7)	10,822 (56.5)	3062 (52.3)	19,136 (50.9)	33,020 (52.7)
Male	8358 (43.6)	18,496 (49.2)	26,854 (47.3)	8348 (43.5)	2795 (47.7)	18,493 (49.1)	29,636 (47.3)
*Level of education*							
Primary	3487 (18.2)	7874 (21.2)	11,361 (20.2)	3476 (18.1)	2274 (38.8)	7875 (21.2)	13,625 (21.9)
Secondary	11,206 (58.4)	20,855 (56.3)	32,061 (57.0)	11,201 (58.4)	2857 (48.8)	20,853 (56.3)	34,911 (56.2)
Tertiary	4492 (23.4)	8334 (22.5)	12,826 (22.8)	4491 (23.4)	725 (12.4)	8332 (22.5)	13,548 (21.8)
*Wealth*							
1st Quintile	3187 (16.6)	6268 (16.7)	9455 (16.7)	3182 (16.6)	906 (16.4)	6268 (16.7)	10,356 (16.7)
2nd Quintile	3665 (19.1)	6945 (18.5)	10,610 (18.7)	3658 (19.1)	928 (16.8)	6943 (18.5)	11,529 (18.6)
3rd Quintile	4031 (21.0)	7376 (19.7)	11,407 (20.1)	4030 (21.0)	1427 (25.9)	7377 (19.7)	12,834 (20.7)
4th Quintile	4084 (21.3)	8220 (21.9)	12,304 (21.7)	4082 (21.3)	1067 (19.4)	8220 (22.0)	13,369 (21.5)
5th Quintile	4220 (22.0)	8640 (23.1)	12,860 (22.7)	4218 (22.0)	1182 (21.5)	8639 (23.1)	14,039 (22.6)
*Current smoking*							
No	16,160 (84.6)	30,417 (80.8)	46,577 (82.1)	16,146 (84.6)	4683 (80.0)	30,415 (80.8)	51,244 (81.9)
Yes	2943 (15.4)	7209 (19.2)	10,152 (17.9)	2940 (15.4)	1173 (20.0)	7209 (19.2)	11,322 (18.1)
*Current alcohol drinking*							
No	8254 (43.0)	9962 (26.5)	18,216 (32.1)	8245 (43.0)	3492 (59.6)	9964 (26.5)	21,701 (34.6)
Yes	10,930 (57.0)	27,664 (73.5)	38,594 (67.9)	10,922 (57.0)	2365 (40.4)	27,660 (73.5)	40,947 (65.4)
*Participation in PA*^*d*^							
Never	2882 (15.0)	2683 (7.1)	5565 (9.8)	10,359 (54.0)	3387 (57.8)	13,097 (34.8)	26,843 (42.8)
Once a week	5252 (27.4)	6737 (17.9)	11,989 (21.1)	3588 (18.7)	223 (3.8)	9291 (24.7)	13,102 (20.9)
Twice or more a week	11,053 (57.6)	28,211 (75.0)	39,264 (69.1)	5223 (27.2)	2247 (38.4)	15,241 (40.5)	22,711 (36.2)
*Healthy ageing status*							
1st Quartile	5345 (27.9)	4578 (12.2)	9923 (17.5)	5343 (27.9)	534 (9.1)	4579 (12.2)	10,456 (16.7)
2nd Quartile	4811 (25.1)	8479 (22.5)	13,290 (23.4)	4801 (25.0)	3083 (52.6)	8480 (22.5)	16,364 (26.1)
3rd Quartile	4121 (21.5)	12,335 (32.8)	16,456 (29.0)	4114 (21.5)	883 (15.1)	12,332 (32.8)	17,329 (27.7)
4th Quartile	4910 (25.6)	12,239 (32.5)	17,149 (30.2)	4912 (25.6)	1357 (23.2)	12,238 (32.5)	18,507 (29.5)

The data correspond to the first record of each participant. ^a^SHARE refers to the Survey of Health Ageing and Retirement in Europe. ^b^HRS refers to the Health and Retirement Study. ^c^KLOSA refers to Korean Longitudinal Study. ^d^PA refers to physical activity

From the 62,656 participants, 11,717 (HRS: 8,014, KLOSA: 3,251, SHARE: 452) developed depression at some point, and 8,032 developed depression in the first follow-up after baseline (HRS: 6003, KLOSA: 1797, SHARE: 232).

Table 2 presents the IRR of depression according to physical activity in light-to-moderate or vigorous intensity. Compared to never, light-to-moderate physical activity, independent of the frequency, was associated with lower IRR of depression (once/week: 0.614, 95% CI 0.586–0.643; twice or more/week: 0.466, 95% CI 0.447–0.486). Light-to-moderate physical activity continued to be associated with lower IRR of depression even after considering the healthy ageing status (once/week: 0.766, 95% CI 0.732–0.802; twice or more/week: 0.683, 95% CI 0.656–0.711).

**Table 2:** Longitudinal association between levels of physical activity (light-to-moderate and vigorous intensities) with depression in older adults

Incidence risk ratio of incident depression (95% confidence intervals)		
	PA^a^ + confounders model	PA^a^ + confounders + healthy ageing model
*Participation in light-to-moderate PA*		
Once a week versus never	0.614 (0.586–0.643)	0.766 (0.732–0.802)
Twice or more a week versus never	0.466 (0.447–0.486)	0.683 (0.656–0.711)
*Participation in vigorous PA*		
Once a week versus never	0.667 (0.637–0.697)	0.848 (0.811–0.887)
Twice or more a week versus never	0.594 (0.569–0.620)	0.830 (0.795–0.866)

^a^Refers to physical activity. Confounders: sex, age, education level, wealth, current smoking, current alcohol drinking and cohort study

Compared to never, vigorous physical activity, independent of the frequency, was associated with lower IRR of depression (once/week: 0.667, 95% CI 0.637–0.697; twice or more/week: 0.594, 95% CI 0.569–0.620). Vigorous physical activity continued to be associated with lower IRR of depression even after considering the healthy ageing status (once/week: 0.848, 95% CI 0.811–0.887; twice or more/week: 0.830, 95% CI 0.795–0.866).

Additional file 1: Table S3 presents the association between physical activity participation (light-to-moderate and vigorous intensities) classified in four categories (never, once a week, 2–3 days/week and more than three days/week) and depression.

**Fig. 2:**
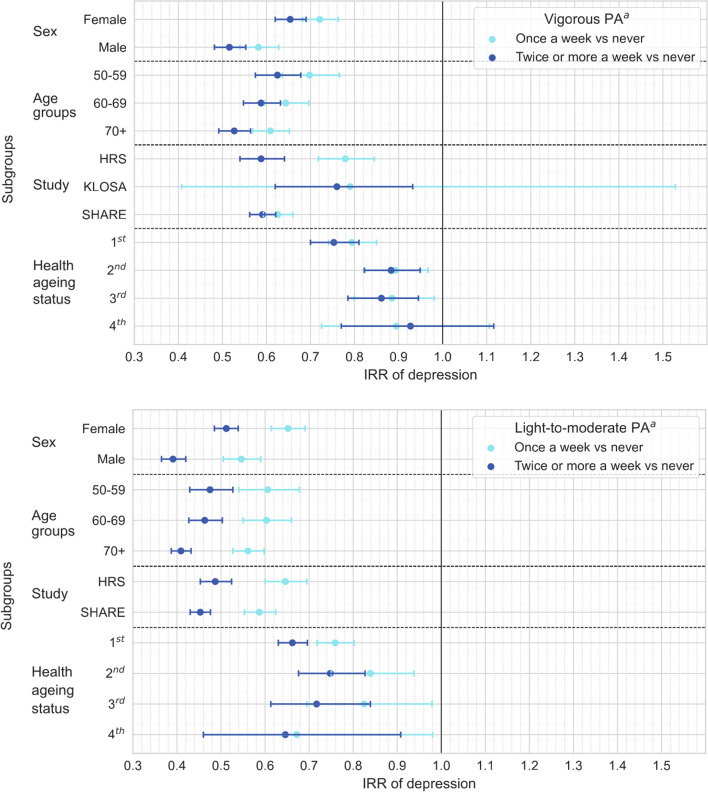
Longitudinal association between levels of physical activity (light-to-moderate and vigorous intensities) with depression in older adults by sex (male and female), age group (50–59, 60–69, 70+ years), study (HRS, KLOSA and SHARE) and the ATHLOS healthy ageing status at baseline (1st to 4th quartile). Data presented as incidence risk ratio and respective 95% confidence intervals

Figure 2 and Additional file 1: Table S4 present the longitudinal association between physical activity and depression stratified by sex, age group, study cohort and healthy ageing status. In summary, light-to-moderate or vigorous physical activity, in any weekly frequency, protected against incident depression regardless of sex or age group (50–59, 60–69 and ≥ 70 years). Participants from all studies who participated in physical activity of any intensity and weekly frequency exhibited lower IRR of depression, except for participants in the KLOSA study to whom vigorous physical activity needed a frequency of at least twice/week to protect against depression. Physical activity of any intensity and weekly frequency protected against depression despite participants’ healthy ageing quartile classification, except for the ‘healthiest’ participants (4th quartile) in which vigorous physical activity was not associated with lower IRR of depression.

## Discussion

Using a harmonised dataset of ageing cohorts from the USA, the Republic of Korea and Europe and Israel comprising more than 20 countries and 62,656 (vigorous models) and 56,818 (light-to-moderate models) participants, we observed that physical activity of any intensity and weekly frequency was strongly associated with lower risk of depression in older adults. In particular, participating in light-to-moderate or vigorous physical activity at least once a week was already a strong protector against depression in older adults. Furthermore, higher physical activity frequency was related to even stronger protection against depression. Nevertheless, 2–3 times/week of physical activity entailed equivalent protection against depression compared to higher frequency, regardless of intensity.

We evaluated the physical activity–depression relationship considering an indicator for healthy ageing that comprises multiple domains of health and functioning across cohorts and monitoring periods. The healthy ageing concept represents the physical and mental functionalities of a person instead of focusing on symptoms and pathological abnormalities that might be present in an older person, which has been the focus of other relevant but distinct concepts such as frailty [18]. Importantly, our findings demonstrate that physical activity of any intensity or weekly frequency is a strong determinant of depression in older persons despite their healthy ageing scale, establishing the relevance of physical activity despite the person’s intrinsic capacity and functional ability.

Our findings indicated that physical capacity, portrayed by the healthy ageing scale, is not the only mechanism explaining the relationship of physical activity with depression. Biological (neuroplasticity, inflammation, oxidative stress and the neuroendocrine system) and psychosocial (self-esteem, social support and self-efficacy) parameters are relevant in the physical activity–depression relationship [19]. Although the biggest wealth of studies indicate a higher importance for biological factors [19], the fact that light-to-moderate or vigorous physical activity exhibited similar strength of association in terms of the risk of depression suggests that psychosocial parameters might be even more relevant. While higher physical activity intensities ignite biological changes that are related to lower levels of depression [6, 8, 19–21], biological responses are less pronounced in low-intensity physical activity [22, 23]. On the other hand, psychosocial factors are presented in all intensities, especially in activities performed in groups [19].

Physical activity conferred stronger protection against depression in men, although physical activity also lowered the risk of depression in women. Men are more likely to participate in physical activities [24]; therefore, it is possible that men showed a lifetime accumulated protection against depression. Physical activity, of any intensity and weekly frequency, exhibited strong protection against depression regardless of the age group (50–59, 60–69, ≥ 70 years), although the older population, generally, presented the highest benefits compared to their younger peers. The older population who participated in physical activity might have engaged in physical activities during their lifetime, conferring an accumulated physical activity protection against depression in this group [20].

We observed subtle differences in the strength of the physical activity–depression association across cohort studies. The association in the KLOSA (Republic of Korea) cohort was slightly lower compared to the HRS (the USA) and the SHARE (Europe and Israel) cohorts. These differences might be related to cultural differences and stigma towards mental health [25]. Participants, especially in Asia, might have distinct perceptions on their mental health, and likely a higher stigma on the topic, which could have affected their answers to the questionnaires and subsequent depression classification. Nevertheless, a frequency of twice or more/week of vigorous physical activity protected against depression in the KLOSA cohort compared to no participation.

Interestingly, physical activity seems to confer the biggest protection towards depression to older adults with the poorest healthy ageing score. Older adults with poor health are more likely to develop depression and other health outcomes [26, 27], and thus, our findings are particularly relevant to the design of public health policies. Physical activity promotion is one of the Best Buys programmes of the World Health Organization to tackle non-communicable diseases because of its cost-effectiveness and societal benefit [28], and successful scale up evidence [29]. Our results highlight the importance of promoting physical activity especially to population groups at higher risk of having poor overall health (socioeconomically, minorities, immigrants, etc.) [30].

## Limitations

Studies in the ATHLOS consortium from low-income and middle-income countries did not contain information on physical activity and depression in at least two waves of data and could not be included in this longitudinal analysis. Despite the process of data harmonisation, variation in methods of data collection or management across cohort studies should still be considered when interpreting the findings. Measures from different studies might collect slightly different information, and thus, variation in measurements might affect the associations evaluated. Further, some societal and historical factors such as health systems, welfare policies or economic crises in different societies might also affect health throughout the lifetime and partly explain our findings. However, we adjusted the analyses for cohort study and conducted stratified analyses observing associations in all cohort studies. Physical activity data were collected from questionnaires, thus compromising the quality of the physical activity assessment compared to direct assessments such as accelerometers. Moreover, the cohort studies providing data to the ATHLOS study did not use established physical activity questionnaires. Nevertheless, the questions asked are very similar to the questions in the IPAQ questionnaire [31]. We did not have information on the domain of physical activity or sedentary behaviour; hence, we were not able to take this into account in our analyses.

## Conclusions

In this cohort study of 62,591 older adults, physical activity of any intensity and weekly frequency, was a strong protective factor against depression, independent of their physical and mental functioning. Further, physical activity protected against depression in all subgroups analysed: females and males, age group (50–59, 60–69 and ≥ 70 years), cohort study or healthy ageing profile—except for older adults with highest healthy ageing score.

Large-scale clinical studies could evaluate the impact of incorporating lower-intensity physical activity in relation to the risk of depression in older adults. Public health policies should stimulate the incorporation of lower physical activity intensities to prevent depression in older adults, which might be more feasible at the population level.

### Supplementary Information

**Additional file 1. **Supplementary information about the methodology of the study and additional results.

## Data Availability

A harmonised dataset can be created for each wave and population according to the ATHLOS DataSchema and the harmonisation codes located on Github: DataSchema: https://athlos.pssjd.org/ws/file-dl/network/athlos/DataSchema_ATHLOS.xlsx Harmonisation codes: https://github.com/athlosproject/athlos-project.github.io/tree/master/HRS; https://github.com/athlosproject/athlos-project.github.io/tree/master/SHARE; https://github.com/athlosproject/athlos-project.github.io/tree/master/KLOSA Anyone interested in using the harmonised data from the HRS study via the ATHLOS DataSchema should register on the website https://g2aging.org/, download the necessary data and use the harmonisation codes. For more detailed help on this process, please contact hrspublications@umich.edu. Anyone interested in using the harmonised data from the SHARE and KLOSA studies via the ATHLOS DataSchema should register on the website http://www.share-project.org/, download the necessary data and use the harmonisation codes.

## Abbreviations

ATHLOS: EU Ageing Trajectories of Health: Longitudinal Opportunities and Synergies consortium
HRS: Health and Retirement Study
SHARE: Survey of Health Ageing and Retirement in Europe
KLOSA: Korean Longitudinal Study
CES-D: Depression Scale of the Center for Epidemiological Studies
EURO-D: European Union initiative to compare symptoms of depression
IRT: Item response theory
GEE: Generalised estimating equation
IRR: Incidence rate ratio
QIC: Quasi-information criterion
CIC: Correlation information criterion
