# Supplementary material for: Physical Activity Participation Decreases the Risk of Depression in Older Adults: The ATHLOS Population-Based Cohort Study
Source: Sports Med Open. 2024 Jan 3;10:1. doi: 10.1186/s40798-023-00664-7 (PMC10764659; doi:10.1186/s40798-023-00664-7)
Supplement: Supplementary file 1 — Additional file 1. Supplementary information about the methodology of the study and additional results. [file 40798_2023_664_MOESM1_ESM.docx]

**Physical activity participation decreases the risk of depression in older adults: the ATHLOS population-based cohort study**

**Sports Medicine**

**Authors:** Rodrigo A Lima, PhD^1,2^, Elena Condominas, MSc^1,2^, Albert Sanchez-Niubo, PhD^1,2,3^, Beatriz Olaya, PhD ^1,2^, Ai Koyanagi, PhD ^1,2,4^, Carlota de Miquel, MSc^1,2^, Josep Maria Haro, PhD^1,2^

**Affiliations:**

^1^Research, Innovation and Teaching Unit, Parc Sanitari Sant Joan de Déu, Sant Boi de Llobregat, Spain

^2^Centro de Investigación Biomédica en Red de Salud Mental (CIBERSAM), Instituto de Salud Carlos III, Madrid, Spain.

^3^Department of Social Psychology and Quantitative Psychology, University of Barcelona, Barcelona, Spain.

^4^ICREA, Pg. Lluis Companys 23, 08010, Barcelona, Spain

Corresponding author: Rodrigo A Lima, Dr Antoni Pujadas 42, Sant Boi de Llobregat, Barcelona 08830, +34 611 14 03 67; rodrigoantlima@gmail.com

**Supplementary Online Content**

**Supplementary Table 1.** List of 41 intrinsic capacity and functional ability items included in the ATHLOS healthy ageing scale.

| **Domain** | **Variables** |
| --- | --- |
| Cognition | Memory  Immediate recall  Delayed recall  Verbal fluency  Orientation in time  Processing speed  Numeracy |
| Psychology symptoms | Sleeping |
| Vitality | Experiences some degree of pain  Having high level of energy |
|  | Urinary incontinence |
| Sensory functions | Near vision  Far vision  Eyesight using glasses or lens as usual  Hearing in general  Hearing in a conversation |
| Locomotion/mobility | Stooping, kneeling or crouching  Lifting or carrying weights  Climbing stairs  Getting up from sitting down  Walking by yourself and without any equipment  Pulling or pushing large objects  Sitting for long periods  Reaching or extending arms  Walking speed  Dizziness when walking on a level surface  Picking up things with fingers |
| Activities of daily living | Getting in or out of bed  Bathing or showering  Getting dressed  Moving around the home  Using the toilet  Eating |
| Instrumental activities of daily living | Doing housework  Shopping for groceries  Getting out of the house  Difficulties in preparing meals  Using a map  Managing money, bills or expenses  Taking medications  Making telephone calls |

**Supplementary Figure 1.** Timeline of the cohort studies and respective waves that contributed with data to the current investigation.

**Legend:** ^a^HRS: Health and Retirement Study; ^b^KLOSA: Korean Longitudinal Study; ^c^SHARE: Survey of Health Ageing and Retirement in Europe.

The HRS is a US national longitudinal survey conducted every second year since 1992 in 23,000 households with people older than 50 years of age[1,2]. From the HRS study, we used harmonised data based on five waves – waves 7 to 11 (between 2004 and 2012). On average, HRS participants were monitored in 3.73 (±1.33) waves.

The SHARE study followed older adults usually every second year since 2004 from the European Union and Israel[3–12]. From the SHARE study, we used harmonised data based on the waves 1 to 5 (DOIs: 10.6103/SHARE.w1.260[13], 10.6103/SHARE.w2.260[14], 10.6103/SHARE.w3.100[15], 10.6103/SHARE.w4.111[16], 10.6103/SHARE.w5.100[17], respectively). On average, SHARE participants were monitored in 2.49 (±0.74) waves.

KLOSA is a national longitudinal study with biennial surveys since 2006 until 2012 that monitored people aged 45 years or older in the Republic of Korea (South Korea)[18]. From the KLOSA study, we used harmonised data based on waves 1 to 4. On average, KLOSA participants were monitored in 3.54 (±0.74) waves.

**Additional description of the Poisson** **generalised estimating equation (GEE) mixed model**

All the Poisson GEE mixed-models applied to evaluate the association between physical activity and depression included all the information in the outcome, exposures and confounders available of a participant. In particular, the IRR of depression presented is based on the physical activity participation in a particular wave where the participant contributed with data and the relative risk of depression in a subsequent wave. For example, for a participant in a particular study with information in waves 1 (baseline), 2 and 5, the IRR of depression for this person is the average of the risk of depression at waves 2 and 5 based on the physical activity participation at waves 1 and 2, respectively. This estimation is conducted for all individuals until the IRR between physical activity and depression is calculate for all participants in every wave that they contributed with data, hence the population-averaged IRR is estimated. Note that the model also considered the confounders and the healthy ageing scale in every wave with data in the example given. Furthermore, the model accounts for the longitudinal nature of the data, thus considering the interdependency of a variable (exposures or outcome) in a subsequent wave based on the information from the previous wave.

**Below the STATA code used for the main analysis (presented in Table 2) with pertinent clarification when needed.**

xtset athlos_id2 new_wave // Setting the dataset for the Poisson generalised estimating equation (GEE) mixed model.

xtgee depression age i.sex i.education i.wealth i.current_smoking i.current_alcohol i.LMPA i.study if depression_base==1, family(poisson) link(log) corr(exchangeable) vce(robust) eform // LMPA in relation to depression adjusted for age, sex, education level, wealth, current smoking, current alcohol drinking and cohort study.

xtgee depression age i.sex i.education i.wealth i.current_smoking i.current_alcohol i.LMPA i.study i.healthstatus if depression_base==1, family(poisson) link(log) corr(exchangeable) vce(robust) eform // LMPA in relation to depression adjusted for age, sex, education level, wealth, current smoking, current alcohol drinking, cohort study and healthy ageing.

xtgee depression age i.sex i.education i.wealth i.current_smoking i.current_alcohol i.VPA i.study if depression_base==1, family(poisson) link(log) corr(exchangeable) vce(robust) eform // VPA in relation to depression adjusted for age, sex, education level, wealth, current smoking, current alcohol drinking and cohort study.

xtgee depression age i.sex i.education i.wealth i.current_smoking i.current_alcohol i.VPA i.study i.healthstatus if depression_base==1, family(poisson) link(log) corr(exchangeable) vce(robust) eform // VPA in relation to depression adjusted for age, sex, education level, wealth, current smoking, current alcohol drinking, cohort study and healthy ageing.

Variable labels

Study: Refers to cohort study (SHARE (Survey of Health Ageing and Retirement in Europe), HRS (Health and Retirement Study) or KLOSA (Korean Longitudinal Study))

LMPA: Light-to-moderate physical activity

VPA: Vigorous physical activity

depression_base==1 is the command to only include participants without depression at baseline.

**Supplementary Table 2.** Baseline characteristics of study population included in the current study compared to the total in the ATHLOS cohort with information on light-to-moderate and vigorous physical activity.

| Characteristics | Light-to-moderate PA models, No (%) | | | | Vigorous PA models, No (%) | | | |  |
| --- | --- | --- | --- | --- | --- | --- | --- | --- | --- |
|  | **HRS^a^** | | **SHARE^b^** | **Total included** | **HRS** | **KLOSA^c^** | **SHARE** | **Total included** | **Total in ATHLOS** |
|  | No=19187 | | No=37631 | No=56818 | No=19170 | No=5857 | No=37629 | No=62656 | No=238383 |
|  | USA | | Europe |  | USA | South Korea | Europe |  |  |
| Age, means (SD) | 63.7 (10.3) | | 63.2 (9.3) | 63.4 (9.6) | 63.7 (10.3) | 61.0 (9.6) | 63.2 (9.3) | 63.2 (9.6) | 61.2 (12.2) |
| Sex |  | |  |  |  | | | |  |
| Female | 10829 (56.4) | | 19135 (50.8) | 29964 (52.7) | 10822 (56.5) | 3062 (52.3) | 19136 (50.9) | 33020 (52.7) | 124818 (52.4) |
| Male | 8358 (43.6) | | 18496 (49.2) | 26854 (47.3) | 8348 (43.5) | 2795 (47.7) | 18493 (49.1) | 29636 (47.3) | 110116 (46.2) |
| Level of education |  | |  |  |  | | | |  |
| Primary | 3487 (18.2) | | 7874 (21.2) | 11361 (20.2) | 3476 (18.1) | 2274 (38.8) | 7875 (21.2) | 13625 (21.9) | 75144 (31.5) |
| Secondary | 11206 (58.4) | | 20855 (56.3) | 32061 (57.0) | 11201 (58.4) | 2857 (48.8) | 20853 (56.3) | 34911 (56.2) | 107475(45.1) |
| Tertiary | 4492 (23.4) | | 8334 (22.5) | 12826 (22.8) | 4491 (23.4) | 725 (12.4) | 8332 (22.5) | 13548 (21.8) | 40125 (16.8) |
| Wealth |  | |  |  |  | | | |  |
| 1^st^ Quintile | 3187 (16.6) | | 6268 (16.7) | 9455 (16.7) | 3182 (16.6) | 906 (16.4) | 6268 (16.7) | 10356 (16.7) | 39155 (17.6) |
| 2^nd^ Quintile | 3665 (19.1) | | 6945 (18.5) | 10610 (18.7) | 3658 (19.1) | 928 (16.8) | 6943 (18.5) | 11529 (18.6) | 38383 (17.3) |
| 3^rd^ Quintile | 4031 (21.0) | | 7376 (19.7) | 11407 (20.1) | 4030 (21.0) | 1427 (25.9) | 7377 (19.7) | 12834 (20.7) | 41969 (18.9) |
| 4^th^ Quintile | 4084 (21.3) | | 8220 (21.9) | 12304 (21.7) | 4082 (21.3) | 1067 (19.4) | 8220 (22.0) | 13369 (21.5) | 44034 (19.8) |
| 5^th^ Quintile | 4220 (22.0) | | 8640 (23.1) | 12860 (22.7) | 4218 (22.0) | 1182 (21.5) | 8639 (23.1) | 14039 (22.6) | 46834 (21.1) |
| Current smoking |  | |  |  |  | | | |  |
| No | 16160 (84.6) | | 30417 (80.8) | 46577 (82.1) | 16146 (84.6) | 4683 (80.0) | 30415 (80.8) | 51244 (81.9) | 186140 (78.1) |
| Yes | 2943 (15.4) | | 7209 (19.2) | 10152 (17.9) | 2940 (15.4) | 1173 (20.0) | 7209 (19.2) | 11322 (18.1) | 50925 (21.4) |
| Characteristics | **Light-to-moderate PA models, No (%)** | | | | **Vigorous PA models, No (%)** | | | |  |
|  | **HRS^a^** | | **SHARE^b^** | **Total included** | **HRS** | **KLOSA^c^** | **SHARE** | **Total included** | **Total in ATHLOS** |
| Current alcohol drinking |  | |  |  |  | | | |  |
| No | 8254 (43.0) | | 9962 (26.5) | 18216 (32.1) | 8245 (43.0) | 3492 (59.6) | 9964 (26.5) | 21701 (34.6) | 99957 (41.9) |
| Yes | 10930 (57.0) | | 27664 (73.5) | 38594 (67.9) | 10922 (57.0) | 2365 (40.4) | 27660 (73.5) | 40947 (65.4) | 134652 (56.5) |
| Vigorous PA^d^ | |  |  |  |  | | | |  |
| Never | NA | | NA | NA | 10359 (54.0) | 3387 (57.8) | 13097 (34.8) | 26843 (42.8) | 54953 (32.9) |
| Once a week | NA | | NA | NA | 3588 (18.7) | 223 (3.8) | 9291 (24.7) | 13102 (20.9) | 23291 (13.9) |
| Twice or more a week | NA | | NA | NA | 5223 (27.2) | 2247 (38.4) | 15241 (40.5) | 22711 (36.2) | 52863 (31.7) |
| Light-to-moderate PA^d^ | |  |  |  |  |  |  |  |  |
| Never | 2882 (15.0) | | 2683 (7.1) | 5565 (9.8) | NA | NA | NA | NA | 13235 (8.3) |
| Once a week | 5252 (27.4) | | 6737 (17.9) | 11989 (21.1) | NA | NA | NA | NA | 19270 (12.0) |
| Twice or more a week | 11053 (57.6) | | 28211 (75.0) | 39264 (69.1) | NA | NA | NA | NA | 99367 (62.0) |
| Healthy ageing status |  | |  |  |  | | | |  |
| 1^st^ Quartile | 5345 (27.9) | | 4578 (12.2) | 9923 (17.5) | 5343 (27.9) | 534 (9.1) | 4579 (12.2) | 10456 (16.7) | 58472 (24.6) |
| 2^nd^ Quartile | 4811 (25.1) | | 8479 (22.5) | 13290 (23.4) | 4801 (25.0) | 3083 (52.6) | 8480 (22.5) | 16364 (26.1) | 55244 (23.2) |
| 3^rd^ Quartile | 4121 (21.5) | | 12335 (32.8) | 16456 (29.0) | 4114 (21.5) | 883 (15.1) | 12332 (32.8) | 17329 (27.7) | 63108 (26.6) |
| 4^th^ Quartile | 4910 (25.6) | | 12239 (32.5) | 17149 (30.2) | 4912 (25.6) | 1357 (23.2) | 12238 (32.5) | 18507 (29.5) | 60827 (25.6) |

^a^HRS: Health and Retirement Study; ^b^SHARE: Survey of Health Ageing and Retirement in Europe; ^c^KLOSA: Korean Longitudinal Study; and, ^d^PA: physical activity.

**Supplementary Table 3.** Longitudinal association between levels of physical activity (light-to-moderate and vigorous intensities) with depression in older adults.

| **Incidence Risk Ratio of incident depression (95% confidence intervals)** | | |
| --- | --- | --- |
|  | **PA**^a^ **+ confounders model** | **PA**^a^ **+ confounders +**  **healthy ageing model** |
| **Light-to-moderate PA**^a^ |  |  |
| Once a week vs never | 0.646 (0.600 to 0.695) | 0.780 (0.727 to 0.838) |
| Twice or three times a week vs never | 0.481 (0.446 to 0.518) | 0.653 (0.607 to 0.702) |
| More than three times a week vs never | 0.518 (0.460 to 0.582) | 0.698 (0.621 to 0.784) |
| **Vigorous PA**^a^ |  |  |
| Once a week vs never | 0.781 (0.720 to 0.847) | 0.944 (0.870 to 1.025) |
| Twice or three times a week vs never | 0.604 (0.554 to 0.658) | 0.791 (0.725 to 0.862) |
| More than three times a week vs never | 0.637 (0.544 to 0.745) | 0.764 (0.649 to 0.899) |

^a^PA: physical activity. Confounders: sex, age, education level, wealth, current smoking, current alcohol drinking and cohort study.

**Supplementary Table 4.** Longitudinal association between levels of physical activity (light-to-moderate and vigorous intensities) with depression in older adults – sub-group analyses by sex, age group, study and healthy ageing status.

| **Subgroups** | **Light-to-moderate PA**^a^ | | **Vigorous PA**^a^ | |
| --- | --- | --- | --- | --- |
|  | Once a week vs never | Twice or more a week vs never | Once a week vs never | Twice or more a week vs never |
| **Sex** |  |  |  |  |
| Female | 0.652 (0.614 to 0.691) | 0.512 (0.485 to 0.539) | 0.721 (0.682 to 0.763) | 0.654 (0.620 to 0.690) |
| Male | 0.546 (0.505 to 0.590) | 0.391 (0.365 to 0.420) | 0.582 (0.540 to 0.628) | 0.516 (0.482 to 0.553) |
| **Age group** |  |  |  |  |
| 50-59 | 0.606 (0.541 to 0.678) | 0.475 (0.429 to 0.527) | 0.698 (0.636 to 0.766) | 0.625 (0.575 to 0.678) |
| 60-69 | 0.603 (0.550 to 0.660) | 0.463 (0.427 to 0.503) | 0.644 (0.596 to 0.696) | 0.588 (0.548 to 0.632) |
| 70+ | 0.561 (0.527 to 0.598) | 0.409 (0.387 to 0.432) | 0.609 (0.569 to 0.652) | 0.527 (0.492 to 0.564) |
| **Study** |  |  |  |  |
| HRS | 0.646 (0.600 to 0.695) | 0.487 (0.453 to 0.524) | 0.779 (0.718 to 0.845) | 0.588 (0.540 to 0.641) |
| KLOSA |  |  | 0.790 (0.408 to 1.528) | 0.760 (0.620 to 0.932) |
| SHARE | 0.587 (0.553 to 0.624) | 0.453 (0.430 to 0.476) | 0.760 (0.620 to 0.932) | 0.591 (0.562 to 0.621) |
| **Healthy ageing status** |  |  |  |  |
| 1^st^ Quartile | 0.759 (0.718 to 0.802) | 0.662 (0.630 to 0.696) | 0.794 (0.741 to 0.850) | 0.753 (0.700 to 0.810) |
| 2^nd^ Quartile | 0.839 (0.750 to 0.938) | 0.748 (0.676 to 0.827) | 0.893 (0.824 to 0.967) | 0.883 (0.822 to 0.949) |
| 3^rd^ Quartile | 0.825 (0.695 to 0.979) | 0.717 (0.613 to 0.839) | 0.885 (0.799 to 0.981) | 0.861 (0.785 to 0.945) |
| 4^th^ Quartile | 0.672 (0.460 to 0.980) | 0.646 (0.460 to 0.908) | 0.895 (0.725 to 1.106) | 0.927 (0.770 to 1.116) |

**Legend:** ^a^PA: physical activity. All models were adjusted by: sex, age, education level, wealth, current smoking, current alcohol drinking and cohort study

**References**

1. Sonnega A, Faul JD, Ofstedal MB, Langa KM, Phillips JWR, Weir DR. Cohort Profile: the Health and Retirement Study (HRS). Int J Epidemiol [Internet]. Int J Epidemiol; 2014 [cited 2022 Mar 8];43:576–85. Available from: https://pubmed.ncbi.nlm.nih.gov/24671021/

2. Health and Retirement Study, (Version O) public use dataset. Produced and distributed by the University of Michigan with funding from the National Institute on Aging (grant number NIA U01AG009740). Ann Arbor, MI. 2015.

3. Börsch-Supan A, Brandt M, Hunkler C, Kneip T, Korbmacher J, Malter F, et al. Data Resource Profile: the Survey of Health, Ageing and Retirement in Europe (SHARE). Int J Epidemiol [Internet]. Int J Epidemiol; 2013 [cited 2022 Mar 8];42:992–1001. Available from: https://pubmed.ncbi.nlm.nih.gov/23778574/

4. Börsch-Supan A, Brugiavini A, Jürges H, Mackenbach J, Siegrist J, Weber G. Health, ageing and retirement in Europe – First results from the Survey of Health, Ageing and Retirement in Europe. Mannheim; 2005.

5. Börsch-Supan A, Jürges H. The Survey of Health, Ageing and Retirement in Europe – Methodology . Mannheim; 2005.

6. Börsch-Supan A, Brugiavini A, Jürges H, Kapteyn A, Mackenbach J, Siegrist J, et al. First results from the Survey of Health, Ageing and Retirement in Europe (2004-2007). Starting the longitudinal dimension. Mannheim; 2008.

7. Börsch-Supan A, Brandt M, Hank K, Schröder M, editors. The individual and the welfare state. Life histories in Europe. . Heidelberg: Springer; 2011.

8. Schröder M. Retrospective data collection in the Survey of Health, Ageing and Retirement in Europe. SHARELIFE methodology. . Mannheim; 2011.

9. Börsch-Supan A., Brandt M, Litwin H, Weber G. Active ageing and solidarity between generations in Europe: First results from SHARE after the economic crisis. Berlin: De Gruyter; 2013.

10. Malter F, Börsch-Supan A. SHARE Wave 4: Innovations & Methodology. Munich; 2013.

11. Börsch-Supan A, Kneip T, Litwin H, Myck M, Weber G. Ageing in Europe - Supporting Policies for an Inclusive Society. Berlin; 2015.

12. Malter F, Börsch-Supan A. SHARE Wave 5: Innovations & Methodology. . Munich; 2015.

13. Börsch-Supan A. Survey of Health, Ageing and Retirement in Europe (SHARE) Wave 1. Release version: 2.6.0. SHARE-ERIC. Data set. 2013.

14. Börsch-Supan A. Survey of Health, Ageing and Retirement in Europe (SHARE) Wave 2. Release version: 2.6.0. SHARE-ERIC. Data set. . 2013.

15. Börsch-Supan A. Survey of Health, Ageing and Retirement in Europe (SHARE) Wave 3 – SHARELIFE. Release version: 1.0.0. SHARE-ERIC. Data set. 2010.

16. Börsch-Supan A. Survey of Health, Ageing and Retirement in Europe (SHARE) Wave 4. Release version: 1.1.1. SHARE-ERIC. Data set. 2013.

17. Börsch-Supan A. Survey of Health, Ageing and Retirement in Europe (SHARE) Wave 5. Release version: 1.0.0. SHARE-ERIC. Data set. 2015.

18. Park JH, Lim S, Lim J-Y, Han M-K, Yoon IY, Kim J-M, et al. An Overview of the Korean Longitudinal Study on Health and Aging. Psychiatry Investig [Internet]. 2007 [cited 2022 Mar 8];4:84–95. Available from: https://www.researchgate.net/publication/233735826
